# Receptor binding protein of prophage reversibly recognizes the low-molecular weight subunit of the surface-layer protein SlpA in *Clostridioides difficile*

**DOI:** 10.3389/fmicb.2022.998215

**Published:** 2022-10-14

**Authors:** Tanaporn Phetruen, Sittinan Chanarat, Tavan Janvilisri, Matthew Phanchana, Sitthivut Charoensutthivarakul, Wichuda Phothichaisri, Surang Chankhamhaengdecha

**Affiliations:** ^1^Department of Biochemistry, Faculty of Science, Mahidol University, Bangkok, Thailand; ^2^Laboratory of Molecular Cell Biology, Center for Excellence in Protein and Enzyme Technology, Faculty of Science, Mahidol University, Bangkok, Thailand; ^3^Department of Molecular Tropical Medicine and Genetics, Faculty of Tropical Medicine, Mahidol University, Bangkok, Thailand; ^4^Faculty of Science, School of Bioinnovation and Bio-Based Product Intelligence, Mahidol University, Bangkok, Thailand; ^5^Faculty of Science, Excellent Center for Drug Discovery (ECDD), Mahidol University, Bangkok, Thailand; ^6^Department of Biology, Faculty of Science, Mahidol University, Bangkok, Thailand

**Keywords:** bacteriophage, *Clostridioides difficile*, receptor-binding protein, phage tail fiber, surface-layer protein SlpA

## Abstract

Receptor-binding proteins (RBPs) are located at the viral tail and mediate the initial recognition of phage to a specific bacterial host. Phage RBPs have co-evolved with numerous types of host receptors resulting in the formation of a diverse assortment of cognate pairs of RBP-receptors that function during the phage attachment step. Although several *Clostridioides difficile* bacteriophages have been discovered, their RBPs are poorly described. Using homology analysis, putative prophage-tail structure (pts) genes were identified from the prophage genome of the *C. difficile* HN10 strain. Competition and enzyme-linked immunosorbent assays, using recombinant Pts_HN10_M, demonstrated the interaction of this Pts to *C. difficile* cells, suggesting a role as a phage RBP. Gel filtration and cross-linking assay revealed the native form of this protein as a homotrimer. Moreover, truncated variants indicated that the C-terminal domain of Pts_HN10_M was important for binding to *C. difficile* cells. Interaction of Pts_HN10_M was also observed to the low-molecular weight subunit of surface-layer protein A (SlpA), located at the outermost surface of *C. difficile* cells. Altogether, our study highlights the function of Pts_HN10_M as an RBP and potentially paves the way toward phage engineering and phage therapy against *C. difficile* infection.

## Introduction

A receptor binding protein (RBP), located at the tail or distal end of a viral particle, mediates the initial attachment of a bacteriophage to the bacterial host (Nobrega et al., 2018). The RBP possesses a host-recognition module that specifically binds to a unique receptor on bacterial cells (Leiman et al., 2010; Dowah and Clokie, 2018; Dunne et al., 2018). Phage infection typically begins with a reversible binding of the viral tail fiber to the bacterial cell surface. Phage that reversibly binds to bacterial surface components can be released as an infectious particle, promoting the search for its specific receptor. Phage association continues with the bacterial cell surface until the specific receptor is located (Adams, 1959; Baptista et al., 2008; Ge et al., 2020). The resulting specific and irreversible attachment induces a conformational change in the distal tail region, resulting in the injection of the phage genome into the bacterial host (Hu et al., 2015; Bertozzi Silva et al., 2016; Nobrega et al., 2018; Ge et al., 2020). The compatibility of phage RBP and host receptor is believed to dictate the success of the phage infection cycle by restricting the host range. Engineering RBPs using biochemical and structural insights, as well as the principle of phage-host interaction, has extended the bacteriophage host range (Ando et al., 2015; Latka et al., 2021). For example, an engineered *Listeria* phage, generated using chimeric RBPs with the head, neck, and shoulder domains heterologously shuffled, can infect both natural and other *Listeria* hosts (Dunne et al., 2019). In addition, a phagebody cocktail, a library of phage scaffolds with modified host-range determining regions of RBPs, can suppress bacterial resistance to phagebodies across long timescales (Yehl et al., 2019). These studies have highlighted the potential of RBP modification to generate phages with tunable host ranges, allowing targeting of pathogenic bacterial strains.

Customized phage production using engineered RBPs should expand susceptible hosts, including *Clostridioides difficile*. However, a lytic phage has not been described for *C. difficile* and the best characterized temperate phages for this bacteria have a limited host range (Heuler et al., 2021). Clues to the presence of potential RBPs active against *C. difficile* have come from the investigation of diffocin, a phage tail fiber-related protein of the R-type bacteriocins (Gebhart et al., 2015). It was proposed that the diffocin RBP (*rtbM*) is responsible for bacterial cell adhesion. Replacement of *rtbM* with the phage tail-structure M gene (*ptsM*) from a prophage of BI/NAP1/027-type strain resulted in modified diffocins (Avidocin-CDs) capable of killing 16 different *C. difficile* strains of BI/NAP1/027-type (Gebhart et al., 2015). A correlation between sensitivity to a specific Avidocin-CD and ribotype, the polymorphisms of ribosomal RNA genes used for *C. difficile* classification, was identified (Kirk et al., 2017). Although the results support the function of both *rtbM* and *ptsM* as RBP genes of *C. difficile* phage and prophage, respectively, an in-depth analysis of their molecular mechanisms is currently not available.

To understand the biochemical properties and functions of RBPs, we identified novel tail-structure genes of a prophage in the *C. difficile* HN10 genome. This *C. difficile* strain is a phage-inducible host of ΦHN10, a temperate phage with an approximately 250 nm long-myovirus contractile tail that has the broadest susceptible host range among isolated phages (Phothichaisri et al., 2018). The sequence of tail-structure genes harbored by the prophage region in this strain may be a promising candidate for RBP characterization due to its potential broad host range. The organization of tail-structure genes in the prophage was analyzed using a bioinformatic approach. Two structural genes and one putative chaperone were identified based on homology prediction and relative position to tail-structure genes in known prophages. These genes were expressed, purified, and characterized using biochemical assays. Functional investigations using competition and antibody-based assays were also performed. Additionally, an analysis of thermodynamic parameters for the binding between the putative RBP and the low-molecular weight (LMW) surface-layer protein A (SlpA), the most abundant member in the proteinaceous array above the peptidoglycan layer of *C. difficile* (Kirk et al., 2017; Phothichaisri et al., 2018; Dowah et al., 2021; Whittle et al., 2022), suggested a direct intermolecular interaction. This analysis should aid in the understanding of the basis for host recognition of *C. difficile* putative phage RBPs and their receptors, with potential applications in the design of broad host range RBPs for engineering cell-lysis proteins and bacterial detection tools.

## Materials and methods

### Bacterial strains and culture conditions

Eight *C. difficile* reference ribotypes were used in this study, including ribotypes RT 001, RT 012 (630), RT 017, RT 020, RT 023, RT 027 (R20291), RT 046, and RT 056, and which were kindly provided by Prof. Nigel Minton, University of Nottingham. *C. difficile* strains HN2, HN6, HN9, HN10, and HN21 were applied as the susceptible host for ΦHN10 phage according to the previous study (Phothichaisri et al., 2018). *C. difficile* was grown in either brain heart infusion (BHI) medium (Himedia) supplemented with 0.5% yeast extract or TY medium (3% tryptose and 2% yeast extract; Himedia) and incubated in anaerobic condition (10% H_2_, 5% CO_2_, and 85% N_2_) at 37°C (Coy Laboratory Products).

*E. coli* XL10-Gold and Rosetta (DE3) were used as cloning and expression hosts, respectively. They were cultured in Luria-Bertani broth (LB) medium (Himedia) supplemented with 100 μg/ml ampicillin (Amp), 25 μg/ml kanamycin (Km), or 34 μg/ml chloramphenicol (Cm) where appropriate for plasmid maintenance.

### Bacteriophage propagation and purification

The bacteriophage ΦHN10 was obtained from the induction of *C. difficile* strain HN10 with 3 μg/μl mitomycin C (Phothichaisri et al., 2018). The induced phages were propagated through the lytic cycle on the sensitive host *C. difficile* HN21. An aliquot of 10^8^ PFU/ml phages was added to mid-log phase *C. difficile* culture and incubated until the clear culture was observed (around 6 h). Cellular debris was removed by centrifugation at 5,000 × g for 30 min at 4°C, and the supernatant was incubated with 10% PEG-8000 and 1 M NaCl overnight at 4°C. The pellet was collected after centrifugation at 10,000 × g for 30 min at 4°C and resuspended in the SM buffer (10 mM Tris–HCl pH 7.5, 0.15 mM NaCl, 10 mM MgSO_4_) and supplemented with 1 mM CaCl_2_ for further experiments.

### Bioinformatic analysis

Prophage region analysis was run on the PHASTER web server (Arndt et al., 2016). DNA and amino acid sequence alignment of putative RBPs were conducted using Clustal Omega online software (Madeira et al., 2022). The alignment results were visualized using ESPript 3.x (Robert and Gouet, 2014). Protein sequence homology was searched using HHpred (Söding, 2005) and Phyre^2^ (Kelley et al., 2015). The secondary structure of protein sequences was predicted using JPred (Drozdetskiy et al., 2015). The protein structure prediction using AlphaFold2 (Jumper et al., 2021) was run and visualized on ChimeraX (Pettersen et al., 2021).

### Cloning of putative RBPs from HN10 prophage

Genomic DNA of *C. difficile* strain HN10 was extracted using a bacteria DNA extraction kit (Omega Bio-Tek) following the manufacturer’s instructions. The insert DNA sequence was amplified for *in vivo* assembly (IVA) cloning (García-Nafría et al., 2016; Supplementary Table S1). Briefly, a pair of primers were designed to specifically amplify a plasmid. While insert sequences were amplified using primers with an overhang sequence reverse complement with the primers amplifying the vector. The PCR products were treated with *Dpn*I (NEB) to eliminate the original DNA template and co-transformed into *E. coli* XL10-Gold strain. The resultant transformants were selected for sequencing and subsequent protein expression.

### Protein expression and purification

The recombinant plasmids were transformed to the *E. coli* strain Rosetta (DE3) expression host. Protein expression was performed by culturing plasmid-containing bacteria in 0.5 l LB medium to the optical density of 0.6 at 600 nm and induced with 1 mM IPTG. The culture was then incubated for 16 h at 20°C with orbital shaking. Bacterial cells were collected by centrifugation at 8,000 x g for 30 min and resuspended in lysis buffer (50 mM Tris–HCl pH 8.0, 150 mM NaCl, 10 mM imidazole, 0.1% Triton X-100, and 5% glycerol) before disruption by ultrasonication. The soluble cell lysate was obtained by centrifugation at 18,000 x g for 30 min, 4°C, mixed with Ni-NTA affinity resins (Expedeon), and loaded onto a 5-ml Poly-Prep Chromatography Column. The unbound proteins were washed using Tris–HCl buffers with 25 mM imidazole and histidine-tagged protein was eluted from the column using Tris–HCl buffers with 250 mM imidazole. Purified protein was dialyzed against a solution containing 10 mM sodium phosphate buffer pH 7.4 and 150 mM NaCl. The molecular weight and purity of the purified proteins were analyzed using 12% (w/v) discontinuous polyacrylamide gels in Tris-glycine SDS running buffers. Gels were visualized after InstantBlue protein stain (Expedeon).

### Size-exclusion chromatography (SEC) analysis and glutaraldehyde cross-linking

A total of 250 μg of purified proteins were analyzed using SEC analysis on ÄKTApurifier (GE Healthcare) using Superdex™ 200 Increase 10/300 GL column (GE Healthcare). The SEC was operated at 0.4 ml/min using PBS buffer (10 mM phosphate buffer, 140 mM NaCl, pH 7.4) as an eluent. The fractions were collected at 1 ml continuously and concentrated using a speed vacuum concentrator before running on 12% (w/v) discontinuous polyacrylamide gels. The gel filtration standard (Bio-Rad) was used for column calibration and native-protein molecular weight calculation.

In cross-linking experiments, 1 μg of purified proteins was incubated with 1 mM glutaraldehyde (Sigma) in 100 μl of total reaction volume. The reaction was performed in PBS buffer at room temperature. Samples of 20 μl were collected at 5, 10, and 20 min post-incubation followed by adding of 1 M Tris–HCl pH 8.0 for quenching the reaction (North et al., 2019). The cross-linked proteins were analyzed using 10% (w/v) discontinuous polyacrylamide gels.

### Competition assay of phage binding by purified proteins

*C. difficile* was cultured to an optical density of 1 at 600 nm. Cells were harvested by centrifugation at 6,000 × g for 2 min and resuspended with 0.1 volume of culture media to obtain approximately 10^9^ CFU/ml. A concentrated culture of 60 μl was mixed with different concentrations of proteins (5, 10, and 20 μM) and incubated for 20 min at 37°C in anaerobic conditions. Then 10 μl of 10^7^ PFU/ml phage was added and incubated for 5 min at 37°C. A mixture without purified proteins was used as a negative control. The supernatant containing unbound phages was collected after centrifugation at 10,000 × g for 1 min and determined the titer *via* plaque assay (Beck et al., 2009). Counts were compared with a control without purified proteins and shown as percentages of unabsorbed or residual phages to the initial phage titer.

### Enzyme-linked immunosorbent assay

For a whole-cell ELISA, *C. difficile* strains HN2, HN6, HN9, HN21, belonging to ribotype 017, *C. difficile* reference strains, including ribotype 001, 017, 020, 023, 046, 056, 630, and R20291, *C. perfringens*, and *Bacillus subtilis*, were cultured in 96-well MaxiSorp ELISA plates (Thermo Scientific) to mid-log phase, as measured by OD. Then, the culture supernatant was removed, and the plates were blocked with 10% fetal bovine serum (FBS) for 30 min before incubating with 10 μM of purified proteins for 1 h. Three percent skim milk was used for blocking non-specific binding. An anti-histidine-tag rabbit antibody (Cell Signaling Technology) was primarily probed, followed by an HRP conjugated anti-rabbit IgG antibody (Cell Signaling Technology). After TMB (3,3′,5,5″-tetramethylbenzidine) substrate (SeraCare) incubation for 10 min, the signal intensity was recorded at an absorbance of 650 nm using an Infinite 200 PRO microplate reader (Tecan).

For protein ELISA, an assay plate was coated with extracted surface-layer proteins (SLPs) from different *C. difficile* strains on the MaxiSorp plate and incubated at 4°C overnight. The SLPs fraction was extracted from mid-log phase *C. difficile* culture using the low-pH glycine method as described previously (Willing et al., 2015). Briefly, the culture pellet was suspended with 0.2 M glycine pH 2.2 and incubated with gently shaking for 30 min. The SLPs-containing supernatant was collected after centrifugation at 16,000 × g for 15 min and neutralized with 2 M Tris–HCl pH 9.0. The protein concentration of fractions was measured using a standard Bradford assay.

### Isothermal titration calorimetry

ITC experiments were carried out in a buffer of 10 mM Tris–HCl pH 7.4, 100 mM NaCl, and 5% glycerol. Protein was dialyzed against this buffer overnight before experiments and subsequent dilutions of protein and ligands with the remaining dialysis buffer. Generally, ligands at a concentration of around 1–1.25 mM were titrated against a solution of 110 μM Pts_HN10_M using a MicroCal PEAQ-ITC calorimeter (Malvern Panalytical). The running parameters were as follows: 20 ligand injections, 0.2 μl initial injection volume, 2 μl subsequent injection volume, with 90 s between injections, at a reference temperature of 25°C. Data were analyzed using MicroCal PEAQ-ITC analysis software. The titration of an initial injection (0.2 μl) was discarded during data processing.

### Phage inactivation assay

Purified phage at 10^7^ PFU/ml was mixed with different concentrations of purified LMW SlpA and incubated for 30 min at 37°C with gentle agitation. Then, 10 μl of the mixture were taken out for serial dilution and spotted onto BHI plates (Beck et al., 2009). A phage lysate treated with a protein dialysis buffer was used as a negative control. Reversibly adsorbed phages could be released from the putative receptor as infectious particles by dilution, whereas irreversibly bound phages are not recoverable since they become committed to infection (Adams, 1959; Sao-Jose et al., 2006; Baptista et al., 2008).

### Statistical analysis

For statistical analysis, GraphPad Prism 8.4.2 was used. Data from each experiment were checked for normality. After passing the normality test, data were analyzed by ANOVA with *post-hoc* Tukey’s multiple comparison test. Otherwise, data were analyzed using non-parametric ANOVA with *post-hoc* Dunnett’s multiple comparison test.

## Results

### *In silico* analysis revealed the homology of Pts_HN10_ to other known phage proteins

**Figure 1:**
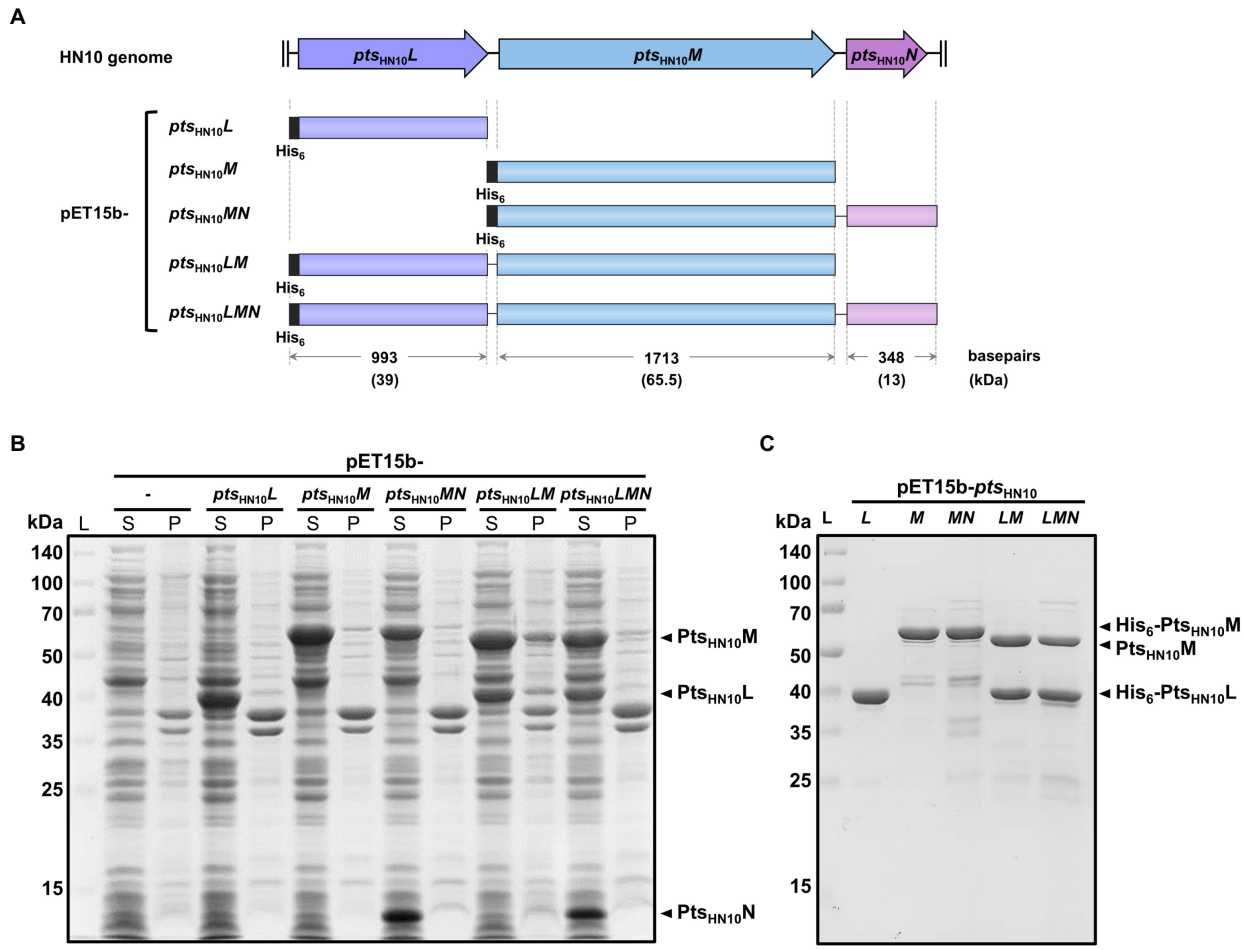
Expression and purification of Pts-related constructs from the HN10 prophage. **(A)** The schematic representation of five constructs from the HN10 prophage. The horizontal boxes indicate the insert sequences covering three genes of interest (*pts*_HN10_*L*, *pts*_HN10_*M*, and *pts*_HN10_*N*). The plasmid nomenclature, sequence length (base pairs), and expected molecular mass of proteins (kDa) with hexahistidine-tag are shown. **(B)** SDS-PAGE analysis shows the total protein expression of five pET15b constructs in *E. coli*. The empty vector of pET15b serves as a control. The expected bands of overexpressed Pts_HN10_L, Pts_HN10_M, and Pts_HN10_N proteins are indicated by black arrowheads. L, protein ladder; S, supernatant; P, pellet. **(C)** The SDS-PAGE analysis demonstrates the purified proteins after Ni-NTA affinity chromatography. The expected bands of Pts_HN10_L and Pts_HN10_M proteins are indicated by black arrowheads. L, protein ladder.

We used the PHASTER application to analyze a putative prophage region in the genome of *C. difficile* HN10 (Arndt et al., 2016), a strain containing temperate myovirus with a broad host range (Phothichaisri et al., 2018). An intact prophage that is potentially inducible to be an infectious phage particle is defined as a region with a PHASTER score above 90. Three intact prophage regions were identified in *C. difficile* HN10 with scores between 120–140. The intact prophage with the highest PHASTER score displayed the general organization of the phage tail-fiber gene cluster, with the RBP-encoding genes placed between the tape measuring protein and the lysis cassette, including the holin and endolysin genes (Habann et al., 2014; Dunne et al., 2018; Kizziah et al., 2020). This intact prophage RBP was further characterized. Three prophage tail-structure (*pts*) genes, *ptsL*, *ptsM*, and *ptsN*, have been identified as tail fiber and RBP-related proteins from *C. difficile* phage and prophages (Gebhart et al., 2015). These three genes from the HN10 prophage were examined for their potential as putative RBPs (Figure 1A).

Amino acid sequence alignment of Pts_HN10_L from the HN10 prophage revealed similarity to the ΦCDMH1 phage and a CD630 prophage, with 77.4 and 49% identity, respectively (Supplementary Figure S1A). Homology-based structure determination of Pts_HN10_L_9–132_ using Phyre^2^ generated a model (approximately 37% of the protein) with similarity to gp105 from *Listeria*-phage A511 with 98.4% confidence (Kelley et al., 2015). Consistent with the Phyre^2^ analysis, the structural prediction of Pts_HN10_L using AlphaFold2 indicated that the N-terminal region could be aligned with the chain B of gp105 (PDB: 6hhk) (Supplementary Figure S1C; Jumper et al., 2021). In the A511 phage, gp105 is proposed to either be involved in tail fiber attachment to the bacterial host or form a part of the tail-fiber network (Habann et al., 2014; Guerrero-Ferreira et al., 2019). Our analysis is consistent with previous reports suggesting that the *ptsL* gene encodes a protein in the baseplate attachment region (BPAR) in *C. difficile* diffocin and prophage (Gebhart et al., 2015). Based on the high sequence homology to other Gram-positive phages including those of *C. difficile*, we hypothesized that the protein encoded by prophage *pts*_HN10_*L* may function either to connect the tail fiber to its network or in host recognition.

Amino acid sequence of Pts_HN10_M showed approximately 54% identity to those of ΦCDMH1 phage and a CD630 prophage (Supplementary Figure S1B). An analysis of Pts_HN10_M against the Pfam database revealed a conserved glycine-rich domain at the N-terminus (Pts_HN10_M_14–267_), although the C-terminal region was more diverse (Supplementary Figure S1B). Glycine-rich regions in certain protein families, such as RNA-binding protein, function as protein-interacting domains (Nawrot et al., 2013). We, therefore, speculated that Pts_HN10_M uses the glycine-rich domain to bind to other baseplate-related proteins. Structure prediction of Pts_HN10_M using AlphaFold2 indicated the presence of an α-helix motif flanked by two β-sandwich motifs at both N- and C-terminus (Supplementary Figure S1D; Jumper et al., 2021). In several phage RBPs, the β-sheet-rich region at the C-terminus functions as a receptor-binding domain for bacterial cell attachment (Dowah and Clokie, 2018; Goulet et al., 2020). In addition, the C-terminus of Pts_HN10_M displays sequence variation which may contribute to host specificity, as shown in some phage RBPs (Spinelli et al., 2006). The *in silico* analysis suggests that Pts_HN10_M is a primary candidate for a phage RBP from *C. difficile* HN10.

In addition to the structural components, the phage tail-fiber assembly often requires a chaperone. In the T4 phage two small chaperones, gp57A and gp38, are needed for proper folding, preventing protein aggregation, and facilitating oligomerization of the long tail-fiber protein gp37 (Galan Bartual et al., 2010; Leiman et al., 2010). While gp38 is absent in the mature T4 particle other phage chaperones could attach to the tail fiber and promote binding to the bacterial surface, such as the tail-fiber chaperone of Mu phage (North et al., 2019). The nearest downstream gene to RBPs usually encodes the phage tail-fiber chaperone (Galan Bartual et al., 2010). We hypothesized that a chaperone may be encoded by *pts*_HN10_*N*, which contains a small open reading frame (ORF) most adjacent to *pts*_HN10_*M*. Both *pts*_HN10_*L* and *pts*_HN10_*M* were selected for further characterization as the putative RBP-encoding and phage tail-fiber chaperone encoding genes.

### Recombinant expression and purification of putative RBPs

To examine biochemical properties and functions of the selected *pts* genes for binding to *C. difficile* (Figure 1A), N-terminally hexahistidine (His_6_)-tagged Pts_HN10_L and Pts_HN10_M were purified using Ni-NTA affinity chromatography. The isolated proteins exhibited the expected sizes of 39 and 65.5 kDa for Pts_HN10_L and Pts_HN10_M, respectively (Figures 1B,C).

We also expressed multiple polycistronic versions of *pts* genes using the T7 promoter of the pET15b vector with a His_6_-tag at the N-terminus of the most upstream gene (Figure 1A). Constructs include *pts*_HN10_*MN*, *pts*_HN10_*LM*, and *pts*_HN10_*LMN*. The polycistronic *pts* genes were utilized to trace the behavior of these proteins as a complex. SDS-PAGE of purified proteins expressed from pET15b-*pts*_HN10_*LM* and *pts*_HN10_*LMN* constructs displayed co-elution of Pts_HN10_M with Pts_HN10_L, indicating that these two proteins form a stable complex (Figure 1C). The presence of Pts_HN10_L in a protein complex is consistent with its annotated function to connect the baseplate subunits with tail fibers in the baseplate attachment region (BPAR) (Gebhart et al., 2015). Although Pts_HN10_N was successfully expressed and could be detected in the soluble fraction of crude lysates (Figure 1B), it was not co-eluted with Pts_HN10_L or Pts_HN10_M and was lost during purification of the protein complexes (Figure 1C). Interestingly, the co-expression of Pts_HN10_M and Pts_HN10_N reduced the formation of protein aggregates when stored at 4°C. Following centrifugation at 10,000 × g for 15 min, the amount of Pts_HN10_M present in the insoluble fraction was reduced by approximately 50% when Pts_HN10_N was co-expressed (Supplementary Figure S2). This suggests a role for Pts_HN10_N as a chaperone capable of promoting the formation of soluble Pts_HN10_M in bacterial cells. This analysis demonstrated that Pts_HN10_L and Pts_HN10_M interact with each other and may form a functional complex. In addition, Pts_HN10_N may function as a chaperone even though it is not present in the Pts_HN10_L/M complex.

### Pts_HN10_L and Pts_HN10_M form oligomers facilitated by the putative chaperone Pts_HN10_N

**Figure 2:**
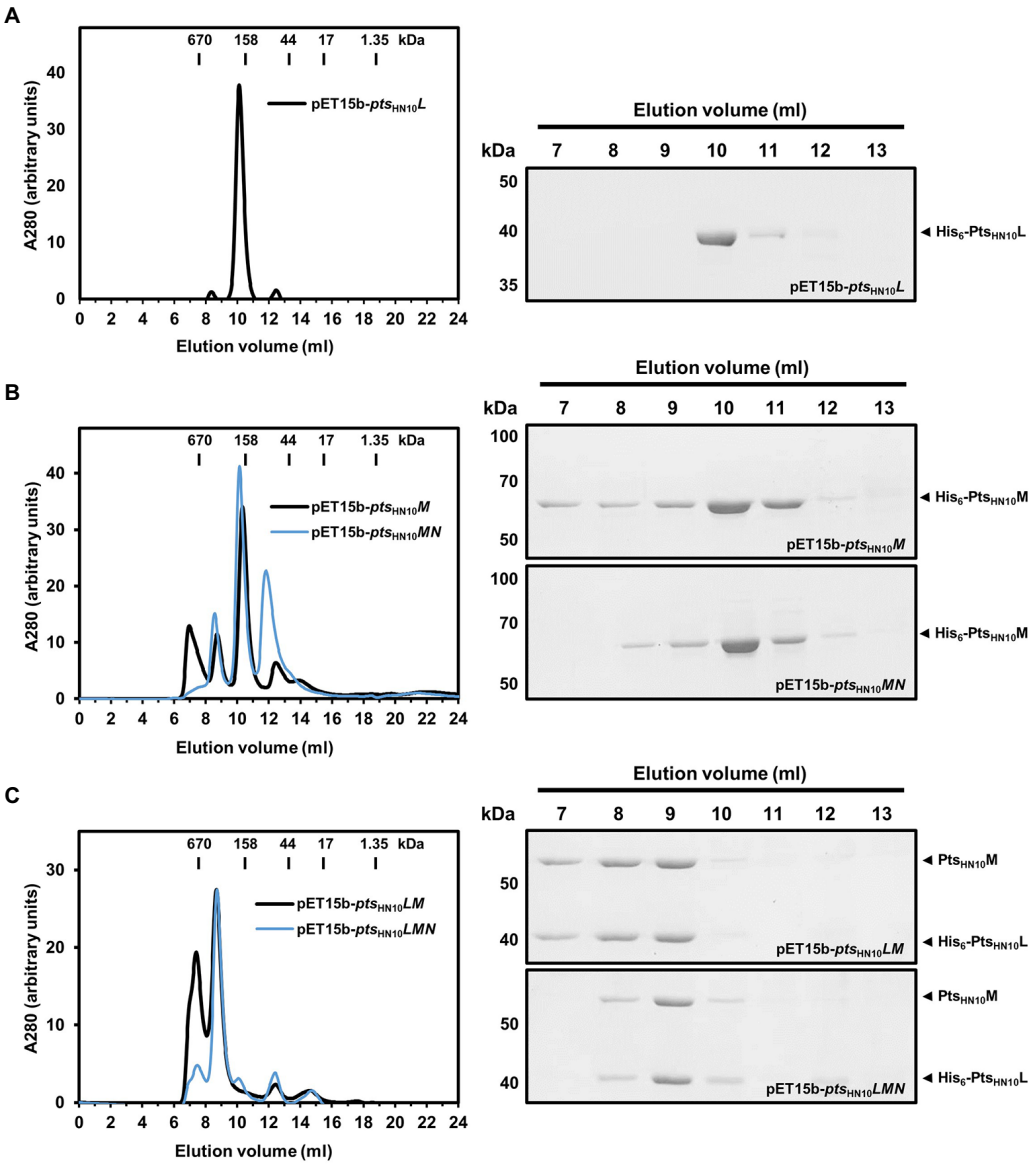
Oligomeric state of putative RBPs in solution. **(A)** Ni-NTA purified proteins expressed from pET15b-*pts*_HN10_*L*, **(B)** pET15b-*pts*_HN10_*M* and *pts*_HN10_*MN* **(C)** pET15b-*pts*_HN10_*LM* and *pts*_HN10_*LMN* constructs were fractionated on a Superdex 200 Increase column. The bars on the size exclusion chromatography graph indicate the molecular weight of standard gel filtration markers. Elution fractions were collected, concentrated, and analyzed by SDS-PAGE. The His_6_-Pts_HN10_L and His_6_-Pts_HN10_M are indicated by black arrowheads.

Under native conditions, most phage tail-fiber proteins are known to oligomerize and form an adsorption apparatus (Dunne et al., 2018). Oligomeric formation of the putative RBPs was analyzed using two complementary methods: size exclusion chromatography (SEC) and chemical cross-linking. In SEC, the proteins were applied to a Superdex 200 Increase column and standard gel-filtration markers were used for molecular mass calibration. The Pts_HN10_L protein was eluted at a fraction corresponding to a molecular mass of 207 kDa. Given that the calculated molecular mass of monomeric Pts_HN10_L with hexahistidine tag is 39 kDa, this result indicated that Pts_HN10_L is forming oligomers in solution (Figure 2A). In the cross-linking experiment, using 5 mM glutaraldehyde, a band on SDS-PAGE of approximately 80 kDa was observed as well as a minor peak (~12 ml) in SEC analysis of Pts_HN10_L protein, corresponding to a Pts_HN10_L dimer (Figure 2A; Supplementary Figure S3A). In summary, Pts_HN10_L may form oligomers and appears to exist predominantly as a homodimer.

Pts_HN10_M fractionated using SEC resulted in the majority of proteins eluting with a retention volume corresponding to a molecular weight of approximately 180 kDa (Figure 2B). A band of approximately 180 kDa was also observed in the chemical cross-linking assay (Supplementary Figure S3B). The calculated molecular mass of monomeric Pts_HN10_M is 65.5 kDa suggesting that Pts_HN10_M is present as a homotrimeric protein, similar to other well-characterized phage RBPs (Dunne et al., 2018; Goulet et al., 2020).

We next investigated whether Pts_HN10_N could stabilize the Pts_HN10_M protein due to its putative chaperone activity (Supplementary Figure S2). Using SEC analysis, it was observed that Pts_HN10_M co-expressed with Pts_HN10_N was eluted with a similar retention volume (~10 ml) as when Pts_HN10_M was expressed alone (Figure 2B). The small peak eluted at ~12 ml corresponding to the expected monomeric species of Pts_HN10_M, with a molecular mass of about 67 kDa. However, a small peak before the column void volume (V_0_ = 7.59 ml), as determined by the elution of thyroglobulin (670 kDa), disappeared when Pts_HN10_M and Pts_HN10_N were co-expressed. The protein eluted at the column void volume is thought to be in a soluble-aggregated form. This finding is consistent with co-expressed Pts_HN10_N promoting the folding or stabilization of the homotrimeric form of Pts_HN10_M during or after bacterial translation, reducing the presence of aggregated Pts_HN10_M.

To evaluate the stoichiometry of the Pts protein complex the molecular mass was determined using SEC analysis. Similar to the results with affinity chromatography, both Pts_HN10_M and Pts_HN10_L proteins were co-eluted using gel filtration chromatography. The estimated molecular mass of the Pts_HN10_L/M complex was ~440 kDa (Figure 2C). SDS-PAGE analysis indicated the ratio of protein intensity in elution fraction was approximately 1:1 (Figure 2C). Given the molecular mass of Pts_HN10_L was around half of Pts_HN10_M, Pts_HN10_L may be twice as abundant as Pts_HN10_M suggesting the molar ratio of 2:1. The calculated molecular weight of the complex from SEC is consistent with a combination of Pts_HN10_L and Pts_HN10_M oligomers both as trimeric species (6 × 39.9 of Pts_HN10_L and 3 × 62.7 of Pts_HN10_M is approximately 440 kDa). It is important to note that co-expression of Pts_HN10_N changed the elution profile of the Pts_HN10_L/M complex during SEC analysis (Figure 2C). The purified Pts_HN10_L/M proteins yielded two major peaks containing both proteins (Figure 2C). In contrast, the presence of Pts_HN10_N during co-expression resulted stabilized the Pts_HN10_L/M complex as one major species resulting in a single elution peak (Figure 2C). Even though Pts_HN10_N did not co-elute with Pts_HN10_L or Pts_HN10_M, these results suggest that Pts_HN10_N may play a role in protein folding or stabilization of the Pts_HN10_L/M complex. Our findings support the hypothesis that Pts_HN10_N is a chaperone for putative tail fibers of HN10 prophage.

### The C-terminus of Pts_HN10_M was necessary for Pts_HN10_L binding

**Figure 3:**
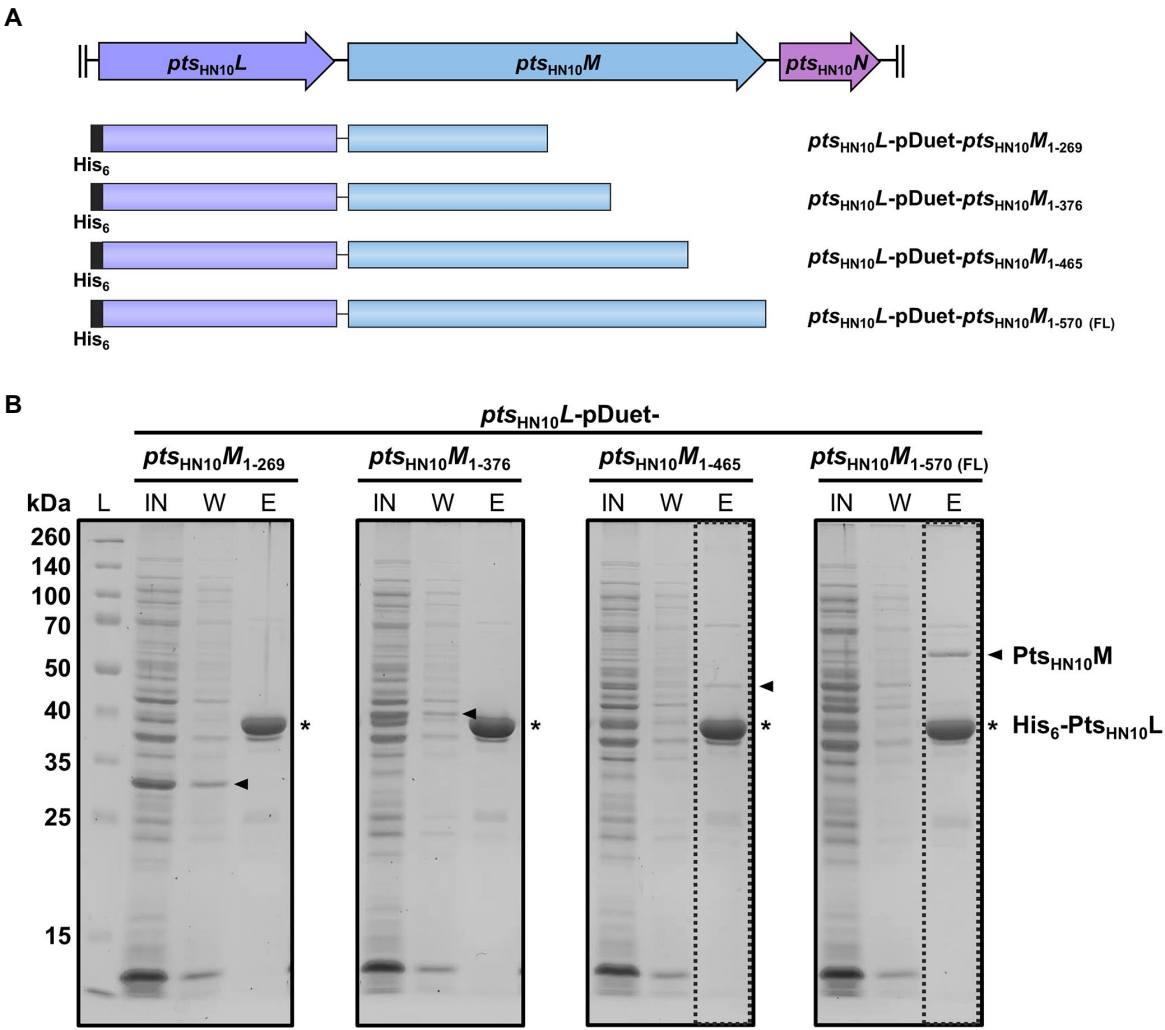
C-terminus of Pts_HN10_M is required for interaction with Pts_HN10_L. **(A)** The schematic representation of four different constructs for co-expression of His_6_-Pts_HN10_L and truncated Pts_HN10_M variants. **(B)** The fractions from protein co-expression purification were collected and visualized using SDS-PAGE followed by Coomassie staining. The bands of His_6_-Pts_HN10_L and truncated Pts_HN10_M proteins are indicated as asterisk and arrowhead, respectively. L, protein ladder; IN, input; W, wash fraction; E, elution fraction. The dashed boxes present an elution fraction that Pts_HN10_M co-elutes with Pts_HN10_L.

Because both affinity and size exclusion chromatography data indicated that Pts_HN10_L and Pts_HN10_M form a complex in solution, we examined the key amino acid residues involved in this interaction. Protein structure prediction using AlphaFold2 and the secondary structure determination using JPred4 revealed two beta-sheet and loop-rich regions at the N- and C-terminus of Pts_HN10_M, while the middle part is consisting of alpha helixes (Drozdetskiy et al., 2015; Jumper et al., 2021). We then used the result from secondary structure prediction to anticipate the deletion of amino acid residues from the C-terminus of Pts_HN10_M, resulting in four distinct constructs (Figure 3A). Co-expression of full-length Pts_HN10_L and C-terminally truncated versions of Pts_HN10_M was used to characterize the Pts_HN10_L-Pts_HN10_M interaction. The *pts*_HN10_*L* gene was cloned into multiple cloning site 1 (MCS1) of pETDuet-1 resulting in a fusion with the N-terminal His_6_-tag. The four truncated *pts*_HN10_*M* variants were cloned into another multiple cloning site of pETDuet-1 without a tag. Recombinant proteins of the four variants were successfully expressed. The interaction between full-length Pts_HN10_L and truncated variants of Pts_HN10_M was examined using affinity chromatography. It was expected that Pts_HN10_L would primarily be in the elution fraction due to the presence of the N-terminal hexahistidine tag. The presence of the Pts_HN10_M truncation variants in the elution fraction would indicate the ability to interact with Pts_HN10_L. The result revealed that both Pts_HN10_M_1–269_ and Pts_HN10_M_1–376_ were in the flow-through fraction indicating a loss of interaction with Pts_HN10_L. Pts_HN10_M_1–465_ and full-length of Pts_HN10_M co-eluted with His_6_-Pts_HN10_L (Figure 3B). Although the glycine-rich region at the N-terminus of Pts_HN10_M is thought to play a role in protein–protein interaction, the co-expression experiments revealed that amino acids 376 to 465 are required for docking Pts_HN10_M to Pts_HN10_L. It is worth noting that the interaction with Pts_HN10_L might not occur at the C-terminus of Pts_HN10_M, but this C-terminal region might be required to stabilize the overall protein structure. We concluded that the C-terminus of Pts_HN10_M is necessary for binding to Pts_HN10_L and for forming the protein complex.

### The C-terminus of Pts_HN10_M is sufficient to bind to *Clostridioides difficile* cells

**Figure 4:**
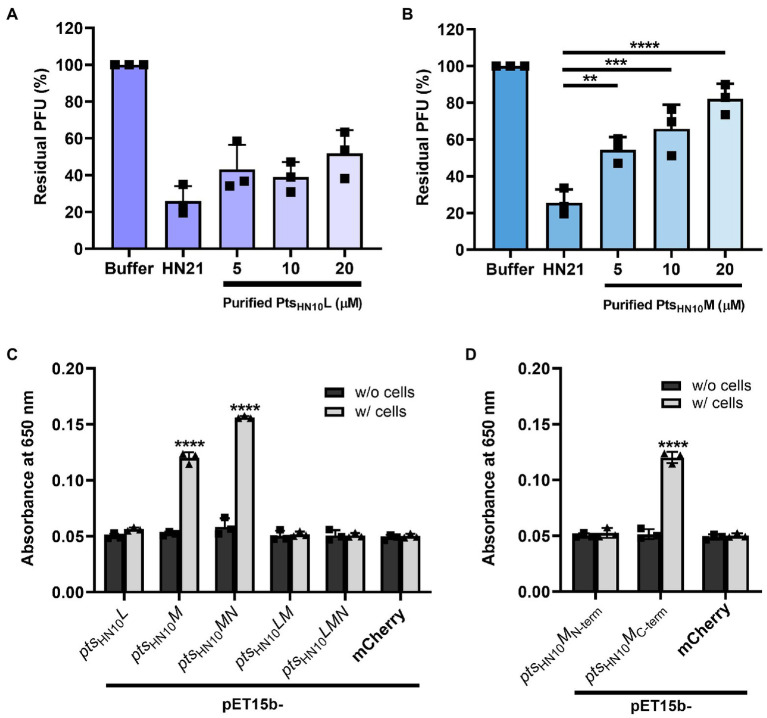
Functional analysis of putative RBPs. The bar graph represents the blockage of phage adsorption by **(A)** purified Pts_HN10_L and **(B)** Pts_HN10_M. Phage titers of each condition were compared with initial phage counts and displayed as percentages of residual plaque-forming unit (PFU). The standard deviation was calculated from three replicates. **(C)** The binding of putative RBPs and *C. difficile* was shown by enzyme-linked immunosorbent assay (ELISA) using purified proteins from five constructs at an absorbance of 650 nm. **(D)** The signal of ELISA using N-terminally and C-terminally truncated variants of PFts_HN10_M and *C. difficile* HN21 strain. Purified mCherry with hexahistidine tag was used as a control for non-specific protein binding. The graphs show the mean and standard deviation from three replicates. The asterisk indicates the significant difference of data (**p < 0.01; ***p < 0.001; ****p < 0.0001) by one-way ANOVA with *post-hoc* Dunnett’s multiple comparison test.

We hypothesized that the selected Pts proteins function in *C. difficile* cell recognition. To establish this interaction, we tested the binding of the two putative RBPs, Pts_HN10_L and Pts_HN10_M, to bacterial cells using two different assays. First, we performed a competition assay to measure the ability of individual proteins to inhibit the adsorption of virion particles onto bacterial cells (McDonnell et al., 2016; Zhang et al., 2018). A basal level of host cell adsorption was measured by incubating fresh cultures of the *C. difficile* HN21 strain, a propagating host of ΦHN10 (Phothichaisri et al., 2018), and phage without the purified proteins. The plaquing efficiency at different concentrations of proteins was measured and calculated as a percentage of residual PFU in the supernatant. We observed that the purified Pts_HN10_L did not compete with the ΦHN10 for adsorption at all tested concentrations (5, 10, and 20 μM) (Figure 4A). In contrast, the purified Pts_HN10_M was capable of competing with ΦHN10 in a concentration-dependent manner, inhibiting phage adsorption to the host bacteria (Figure 4B). Hence, we conclude that Pts_HN10_M alone is sufficient to inhibit ΦHN10 binding to the receptor *in vitro* and may function as the RBP.

Next, we performed a whole-cell enzyme-linked immunosorbent assay (ELISA), which is an antibody-based high-throughput detection method to determine the binding of purified proteins to bacterial cells. As a negative control, we added the purified proteins to the well without bacterial culture. In addition, a histidine-tagged mCherry, a bright red fluorescent protein, was used as a representative of non-specific protein binding to the *C. difficile* HN21 strain. In agreement with the competition assay, the purified Pts_HN10_L did not exhibit a significant increase in signal intensity compared with the negative control (Figure 4C), indicating that Pts_HN10_L could not bind to bacterial cells. Even though purified Pts_HN10_M from pET15b-*pts*_HN10_*M* and *pts*_HN10_*MN* constructs displayed the ability to bind to bacterial cells, the signal intensity of protein expressed from pET15b-*pts*_HN10_*MN* was higher than pET15b-*pts*_HN10_*M*. This indicates that purified Pts_HN10_M functions more effectively when co-expressed with Pts_HN10_N, consistent with the proposed role of Pts_HN10_N as a chaperone. No significant difference in binding to bacterial cells was observed between purified proteins from pET15b*-pts*_HN10_*LM,* pET15b*-pts*_HN10_*LMN* constructs, and the control indicating that the *pts*_HN10_*LM* complex was not able to attach to intact bacteria using this assay. To gain further insight into domains important for *C. difficile* recognition, N-terminal (1–269 AA) and C-terminal (259–570 AA) variants of Pts_HN10_M were constructed. Recombinant proteins were then expressed, purified, and used in ELISA, which revealed that only the C-terminus of Pts_HN10_M was sufficient for binding to bacterial cells (Figure 4D). Of the proteins examined only Pts_HN10_M full-length and its C-terminal variant appears to bind to *C. difficile* cells, suggesting that the C-terminus (259–570 AA) is most likely the host recognition domain of Pts_HN10_M.

### Pts_HN10_M bound to both phage susceptible and unsusceptible *Clostridioides difficile* strains

We investigated the binding specificity of Pts_HN10_M to diverse *C. difficile* strains, including ΦHN10-susceptible and unsusceptible isolates (Phothichaisri et al., 2018). To this end, we performed ELISA by coating 12 different *C. difficile* strains, containing 5 phage-susceptible and 7 phage-unsusceptible strains, onto microtiter plates and exposing them with purified Pts_HN10_M from pET15b-*pts*_HN10_*MN* construct. The purified Pts_HN10_M protein exhibited the binding efficiency for all five phage-susceptible strains (Supplementary Figure S4). Pts_HN10_M also bound to seven phage non-susceptible strains of different ribotypes, suggesting that the binding range of the protein was broader than lysis-capable ΦHN10 particles. Using ELISA with purified Pts_HN10_M as a probe differentiated *C. difficile* from *C. perfringens* (Supplementary Figure S4). Similar results were observed when another Gram-positive bacterium *Bacillus subtilis* was analyzed, indicating that Pts_HN10_M does not cross-react with other bacterial species and appears to bind specifically to *C. difficile*. This broad-spectrum binding and specificity of Pts_HN10_M therefore may allow the use of phage RBPs for bacterial detection.

### Pts_HN10_M bound to LMW subunit of SlpA

**Figure 5:**
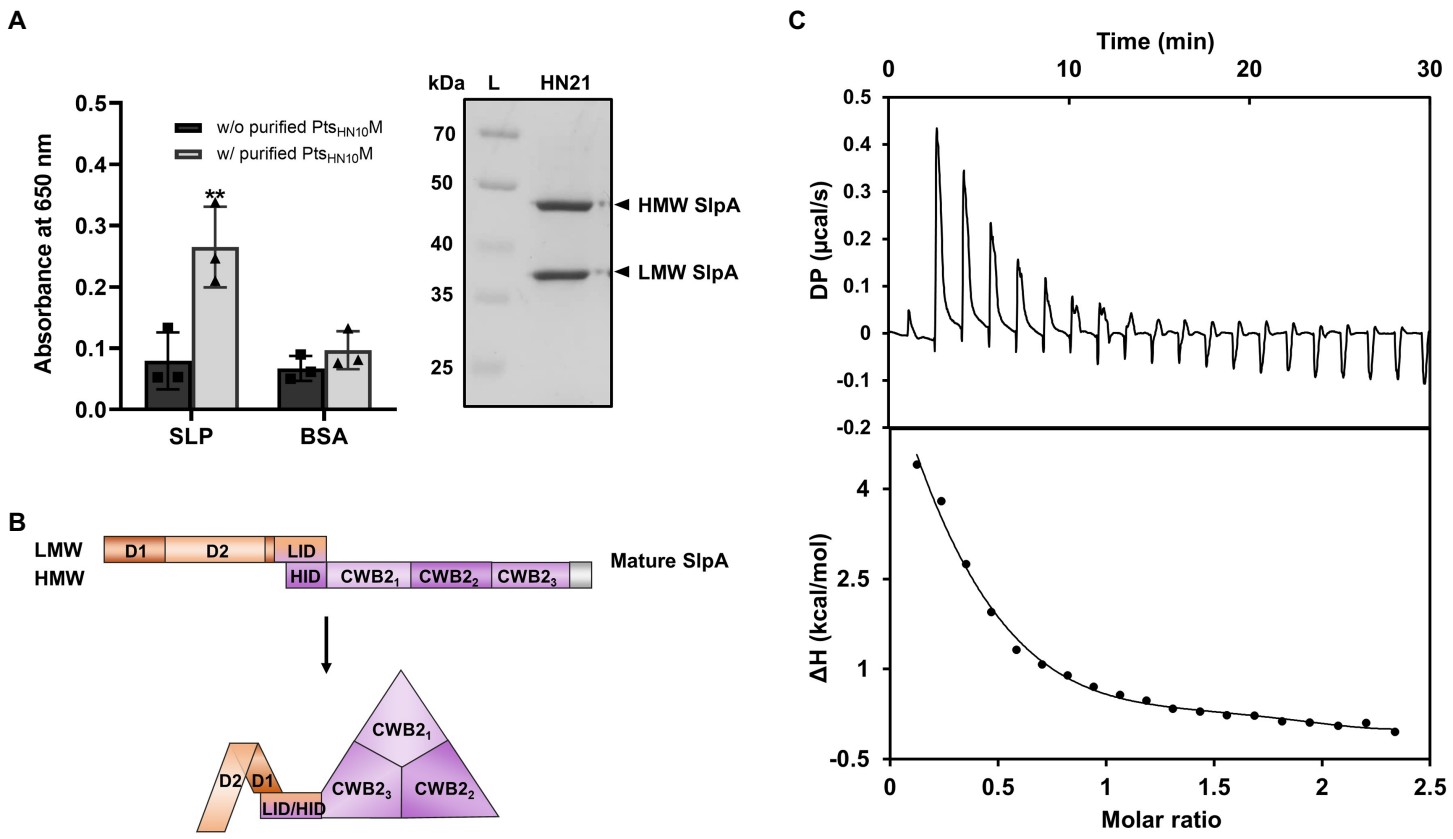
Binding of Pts_HN10_M to SlpA of *C. difficile*. [**(A)**, left] ELISA results of purified His_6_-Pts_HN10_M and low-pH glycine extracted SLP fractions were visualized at an absorbance of 650 nm. The graph shows the mean and standard deviation from three replicates. The asterisk indicates a significant difference in data (*p* < 0.01). [**(A)**, right] SDS-PAGE reveals the SLP fractions extracted from *C. difficile* strain HN21. The bands of high-molecular weight (HMW) and low-molecular weight (LMW) SlpA are indicated. L, protein ladder. **(B)** Schematic representation of the mature SlpA in *C. difficile*. The three CWB2 domains of the HMW form a triangle structure and LMW, especially the D2 domain, is exposed on the exterior of the core CWB2 trimeric complex (Lanzoni-Mangutchi et al., 2022). **(C)** Isothermal titration calorimetry analysis shows thermodynamic parameters of binding between purified Pts_HN10_M and LMW SlpA. The upper panel is the titration raw data for the injection of 0.2–19.8 μl aliquots of 1–1.25 mM ligand solution into 110 μM of protein. The lower panel is the integrated heats of binding obtained from the raw data. The solid line in the bottom panels represent the best curve fit using the one set of sites model.

*C. difficile* naturally expresses a proteinaceous array known as surface-layer protein (SLP) above the thick layer of peptidoglycan. Among 29 cell-wall proteins (CWPs) that decorate the cellular surface of this bacterium, SlpA is the major protein candidate for bacteriophage recognition (Kirk et al., 2017; Phothichaisri et al., 2018; Dowah et al., 2021; Whittle et al., 2022). The SLP fraction extracted from *C. difficile* HN21 strain using the low-pH glycine method exhibited two strong protein bands corresponding to the low-molecular weight (LMW) and high-molecular weight (HMW) subunits of SlpA (Figure 5A; Willing et al., 2015; Phothichaisri et al., 2022). The purified Pts_HN10_M binds to the extracted SLP (Figure 5A) and we suggest that the phage tail-structure protein most likely interacts with SlpA, a putative bacterial receptor and the most abundant protein in the SLP.

The cell-wall binding 2 (CWB2) motif of HMW SlpA is highly conserved; however, the sequence of LMW SlpA is more diverse among *C. difficile* strains (Calabi et al., 2001). The LMW SlpA is composed of two domains, D1 and D2. The high sequence variation is present in the D2 domain and this region is externally exposed from the CWB2 core complex (Fagan et al., 2009; Lanzoni-Mangutchi et al., 2022; Figure 5B). We speculated that the LMW subunit of SlpA might be a specific ligand for Pts_HN10_M, due to its environmental exposure and sequence variation. To examine the role of LMW SlpA in interactions with Pts_HN10_M, we cloned the gene from the *C. difficile* HN21 strain and recombinantly expressed it in *E. coli*. The thermodynamic properties for the binding interaction between the Pts_HN10_M and LMW SlpA were determined by using isothermal titration calorimetry (ITC). This technique facilitates the determination of the protein-ligand interactions in solution without any reporter labeling requirement. A positive titration peak typically indicates that the binding is endothermic (Figure 5C). An enthalpy change (ΔH) of 7.56 kJ mol^−1^ and free energy change (ΔG) of −6.44 kJ mol^−1^ was obtained from the ITC data for the interaction of LMW SlpA and Pts_HN10_M. The negative ΔG suggested that the interaction between Pts_HN10_M and LMW SlpA may be transient, as the dissociation constant (K_D_) of the binding was approximately 19 μM. Determination of the thermodynamic parameters for binding between Pts_HN10_M and HMW SlpA or extracted SLP fraction from *C. difficile* was attempted; however, it was not possible to obtain these results due to the protein aggregation during the preparation steps. Based on our findings, we conclude that a direct interaction between Pts_HN10_M and LMW SlpA was observed and it was thermodynamically favored as the ΔG was below 0.

To examine the type of receptor for LMW SlpA, we next used a phage inactivation assay to differentiate a reversible or primary receptor from an irreversible or secondary receptor, which causes a release of the phage genome and complete inactivation of viral particles (Sao-Jose et al., 2006; Stephan et al., 2020). Different concentrations of purified LMW SlpA were incubated with ΦHN10, the mixture was serially diluted, and the plaquing efficiency was measured (Supplementary Figure S5). According to the competition assay, these concentrations exhibited the ability to block phage adsorption. Therefore, we applied the same concentrations in the phage inactivation assay. However, we observed that the purified LMW SlpA protein was unable to inactivate phage particles at all tested concentrations, implying that the LMW SlpA might not be enough to inactivate phage particles (Supplementary Figure S5). Taken together, our results suggest that the LMW SlpA is a potentially reversible receptor for bacteriophage adsorption.

## Discussion

RBP is one of the most extensively studied proteins among bacteriophage-encoding proteins due to its function in specific host recognition. It has been proposed that the compatibility of RBP and the bacterial host receptor determines the outcome of phage infection. RBP engineering has become a promising strategy for modifying phage to alter the host range (Dunne et al., 2019; Yehl et al., 2019). For *C. difficile*, previous phage RBP studies only examined their effect on bacteriolytic activity (Gebhart et al., 2015; Kirk et al., 2017). In this study, three putative RBP-related genes were identified, and five different combinations of gene constructs were cloned and recombinantly expressed in *E. coli* for biochemical characterization.

Based on our analysis, the Pts_HN10_L is homologous to gp105 or the core part of the baseplate in *Listeria*-phage A511, which was found as 3 units of homodimers in the mature tail-fiber structure (Guerrero-Ferreira et al., 2019). From the homology models, Pts_HN10_L and gp105 appear to share the same oligomeric features. As demonstrated by the cross-linking assay, Pts_HN10_L may form a homodimer, three of which could then form a larger complex based on the molecular mass calculated from size exclusion chromatography (SEC) analysis (approximately 207 kDa or 5.3 times higher than its monomeric unit). The Pts_HN10_M, on the other hand, exhibited a homotrimeric formation, which is a common characteristic of most described phage RBPs. This may reflect the essential nature of the trimeric form for binding to the specific receptor on bacterial cells (Dunne et al., 2018; Goulet et al., 2020). Altogether, our results suggested that Pts_HN10_L and Pts_HN10_M most likely form a dimer and a trimer, respectively. However, it is important to note that due to the limitations of the techniques utilized the oligomerization state of the proteins may need further validation using other methods such as Multi-Angle Light Scattering coupled to size exclusion chromatography (SEC-MALS), X-ray crystallography, or cryogenic electron microscopy (cryo-EM) structure determination.

It is worth noting that, despite the lack of homology between Pts_HN10_N and other bacteriophage proteins, this protein demonstrated an ability to reduce the accumulation of aberrantly folded and aggregated Pts_HN10_M. The difference in SEC profiles of purified Pts_HN10_M from pET15b-*pts*_HN10_*M* and *pts*_HN10_*MN* constructs also reflected the difference in protein folding and decreased aggregation when Pts_HN10_N was co-expressed. In addition, similar results were observed in SEC analysis of purified proteins from pET15b-*pts*_HN10_*LM* and -*pts*_HN10_*LMN,* indicating the role of Pts_HN10_N in complex formation. These results suggest the function of Pts_HN10_N as a chaperone. However, further experiments, such as a luciferase reporter assay, should be performed to verify the activity of Pts_HN10_N in preventing protein aggregation. The separation between the tail fiber protein and its chaperone in the assembled phage structure is similar to that of T4, a well-studied myophage of *E. coli*. In this case, both chaperones including gp57a and gp38 assist the folding of a long tail fiber protein gp37 but are not incorporated in the mature tail structure (Galan Bartual et al., 2010). This information as well as our results highlight the crucial role of phage tail fiber chaperones for proper protein folding.

In both competition assay and enzyme-linked immunosorbent assays (ELISA), purified Pts_HN10_L showed no detectable binding ability. The purified Pts_HN10_M from pET15b-*pts*_HN10_*MN* construct revealed a higher signal intensity than that of pET15b-*pts*_HN10_*M*, implying that co-expression of Pts_HN10_N could prevent the Pts_HN10_M protein from forming the unproductive complex. This result is consistent with the initial observations that co-expression with Pts_HN10_N may reduce protein aggregation. Based on ELISA, the C-terminal region of Pts_HN10_M alone was sufficient to bind to *C. difficile* and suggested that it may contain the host recognition module. In several studies, the N-terminal part of RBP was found to be involved in the attachment of the RBP to other phage tail-fiber components, while the C-terminal part is responsible for receptor binding (Dupont et al., 2004; Hayes et al., 2018; Kizziah et al., 2020). Therefore, the C-terminus of several phage RBPs was the primary target for phage host-range modification (Yoichi et al., 2005; Trojet et al., 2011; Ando et al., 2015; Yosef et al., 2017; Dunne et al., 2019). Considering the results of protein co-expression, the C-terminus of Pts_HN10_M was required for co-elution with Pts_HN10_L, thus indicating the importance of this region for intermolecular interaction between the two Pts proteins. Even though the conserved glycine-rich region in the N-terminal domain of Pts_HN10_M was found to be involved in protein–protein interaction among diverse protein families (Fusaro and Sachetto-Martins, 2007; Nawrot et al., 2013), it is not sufficient to bind to Pts_HN10_L. Our assays, however, had a limitation that conformational changes due to truncations were not examined and the altered structure of the truncated proteins has the potential to limit protein–protein interactions (Goh et al., 2004). Therefore, we interpreted our co-expression results to indicate that the C-terminal domain of Pts_HN10_M is required for the binding to Pts_HN10_L. In conclusion, Pts_HN10_L of HN10 prophage functions to connect tail fiber with other baseplate components and acts as baseplate attachment protein (BPAR) in *C. difficile* phages (Gebhart et al., 2015). Pts_HN10_M indeed functions as RBP, the C-terminus of which contains the host-binding site similar to other described phage RBPs and presumably maintains the structure for interacting with other baseplate-related proteins.

The purified proteins expressed from pET15b-*pts*_HN10_*LM* and *pts*_HN10_*LMN* constructs, on the other hand, showed no detectable binding to bacterial cells. This could be explained based on the cryo-EM structure of the baseplate and tail fibers of the T4 phage which is the most well-investigated myovirus. In the T4 phage, gp12 or the six short tail fibers (STFs) are folded underneath the mature baseplate (Leiman et al., 2004; Yap and Rossmann, 2014). Upon detection of a potential host, the bent region of the STFs then unfolded and straightened along their lengths for host binding. The conformational change in the baseplate is activated by a signal from other proteins in the baseplate complex, such as gp9 that initiates the appropriate orientation of proteins for T4 phage genome delivery (Yap and Rossmann, 2014). Alternatively, an external signal, such as calcium ions, may be essential for baseplate transformation and phage infection in lactococcal phages p2 (Sciara et al., 2010; Spinelli et al., 2020). From our results, it appears that the presence of Pts_HN10_L may cover the host binding site of Pts_HN10_M. The Pts_HN10_L/M complex may require a signal to release and activate Pts_HN10_M for bacterial recognition.

RBP-fused Avidocin-CD is capable of binding to different strains of *C. difficile* (Kirk et al., 2017). Our findings are in agreement with this report as Pts_HN10_M could also bind to both phage susceptible and unsusceptible *C. difficile* strains. The ability to bind a vast range of cells regardless of lytic capability may be explained by the complicated processes of phage infection. Besides adsorption during the initial phage attachment, several steps underlying the success of host cell lysis, including injection of phage genome, phage DNA replication, assembly of phage particles, and other phage resistance mechanisms of bacteria, are also required (Labrie et al., 2010). Hence, the cellular lysis caused by phage infection is not only related to the adsorption ability (Thanki et al., 2018). Interestingly, Pts_HN10_M could distinguish *C. difficile* from other Gram-positive species such as *C. perfringens* and *B. subtilis*, implying that the components of *C. difficile* cell surface recognized by the Pts_HN10_M are not present in other bacterial species. Based on the specificity of Pts_HN10_M, this system has the potential for the development of RBP-based molecular probes for *C. difficile* detection.

To establish the direct interaction between phage RBP and surface receptor of *C. difficile*, we performed ELISA with the extracted SLPs fraction from *C. difficile* culture. In addition, thermodynamic parameters of binding between Pts_HN10_M to LMW SlpA using ITC were determined. The calculated K_D_ was in the micromolar range and the binding reaction was likely transient. Detectable K_D_ from phage RBPs and specific host receptors reported to date range from nanomolar to micromolar. This weak interaction is proposed to be compensated by the cumulative binding of multiple receptors (Dunne et al., 2018). Hence, bacteriophages whose RBP has a weak affinity to host receptors may possess a higher number of RBPs and receptor binding sites. This may increase the affinity of binding and lead to irreversible adsorption. Our results from ITC and the phage inactivation assay suggested that LMW SlpA is most likely a reversible receptor for phage adsorption. In conclusion, we demonstrated that Pts_HN10_M initially contacts LMW SlpA, the outermost surface subunit of SlpA in *C. difficile*, and subsequently Pts_HN10_M or other baseplate-related proteins may irreversibly attach to secondary receptors.

As an alternative treatment for *C. difficile* infection, *C. difficile* phages have limitations due to the lack of a strict lytic phenotype. However, characterization of the putative RBP may expand the knowledge about phage-host interactions for modern phage engineering. As the putative phage RBP of HN10 prophage and its corresponding receptor have been identified, further structural study of phage RBPs and important amino acid residues for host cell recognition could expand phage host range and broaden the application of *C. difficile* phage to both diagnostic and therapeutic purposes.

## Data availability statement

The datasets presented in this study can be found in online repositories. The names of the repository/repositories and accession number(s) can be found at: https://figshare.com/, https://figshare.com/articles/dataset/phage_tail_sequence_fasta/20338716/1.

## Author contributions

TP contributed to idea or design of the research and manuscript writing. SChana contributed to research design, data analysis, and edited the final manuscript. TJ performed the revision and editing of the final manuscript. MP, SChar, and WP helped with the experimental assays. SChank supervised the project, contributed to research design, data analysis, and edited the final manuscript. All authors contributed to the article and approved the submitted version.

## Funding

SChank is supported by the National Research Council of Thailand (NRCT) and Mahidol University Grant NRCT5-RSA63015-26. SChank and TJ are also supported by Research Cluster: Multi-generation Researchers Grant from Mahidol University (MRC-MGR 02/2565) and the East Asia Science and Innovation Area Joint Research Program through the National Research Council of Thailand (NRCT; N10A650693). SChana is supported by the Faculty of Science, Mahidol University and the Research Grant for New Scholars from the Ministry of High Education, Science, Research and Innovation (RGNS 63–178); and WP by Science Achievement Scholarship of Thailand (SAST).

## Conflict of interest

The authors declare that the research was conducted in the absence of any commercial or financial relationships that could be construed as a potential conflict of interest.

## Publisher’s note

All claims expressed in this article are solely those of the authors and do not necessarily represent those of their affiliated organizations, or those of the publisher, the editors and the reviewers. Any product that may be evaluated in this article, or claim that may be made by its manufacturer, is not guaranteed or endorsed by the publisher.
