# Supplementary material for: Receptor binding protein of prophage reversibly recognizes the low-molecular weight subunit of the surface-layer protein SlpA in Clostridioides difficile
Source: Front Microbiol. 2022 Oct 14;13:998215. doi: 10.3389/fmicb.2022.998215 (PMC9615553; doi:10.3389/fmicb.2022.998215)
Supplement: Supplementary file 1 [file Data_Sheet_1.docx]

Supplementary Material

Receptor binding protein of prophage reversibly recognizes the low-molecular weight subunit of the surface-layer protein SlpA in *Clostridioides difficile*

Tanaporn Phetruen^1^, Sittinan Chanarat^1^, Tavan Janvilisri^1^, Matthew Phanchana^3^, Sitthivut Charoensutthivarakul^4,5^, Wichuda Phothichaisri^1^ and Surang Chankhamhaengdecha^6^*

*** Correspondence:** surang.cha@mahidol.edu

# Supplementary Figures and Tables

## Supplementary Figures


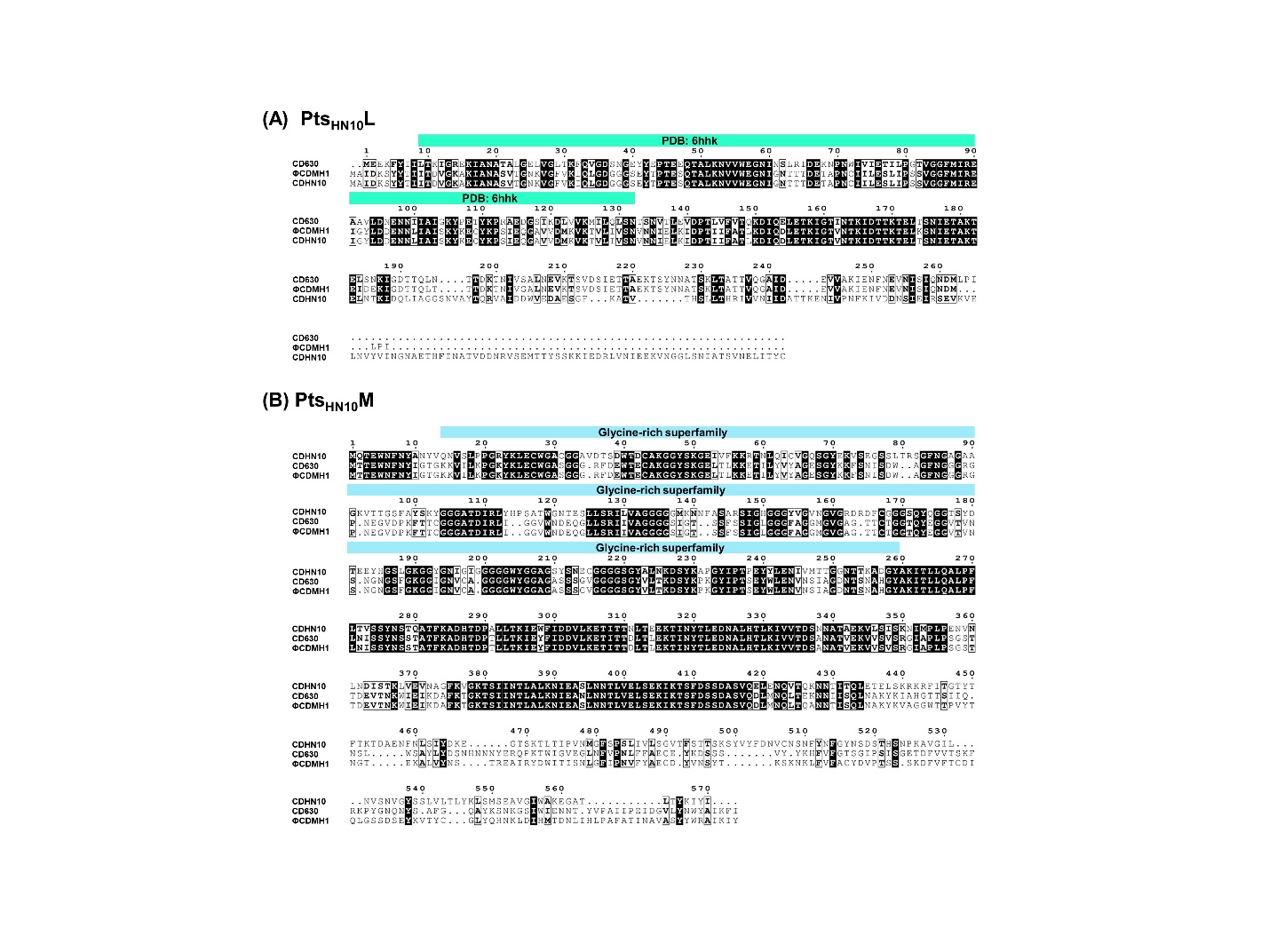


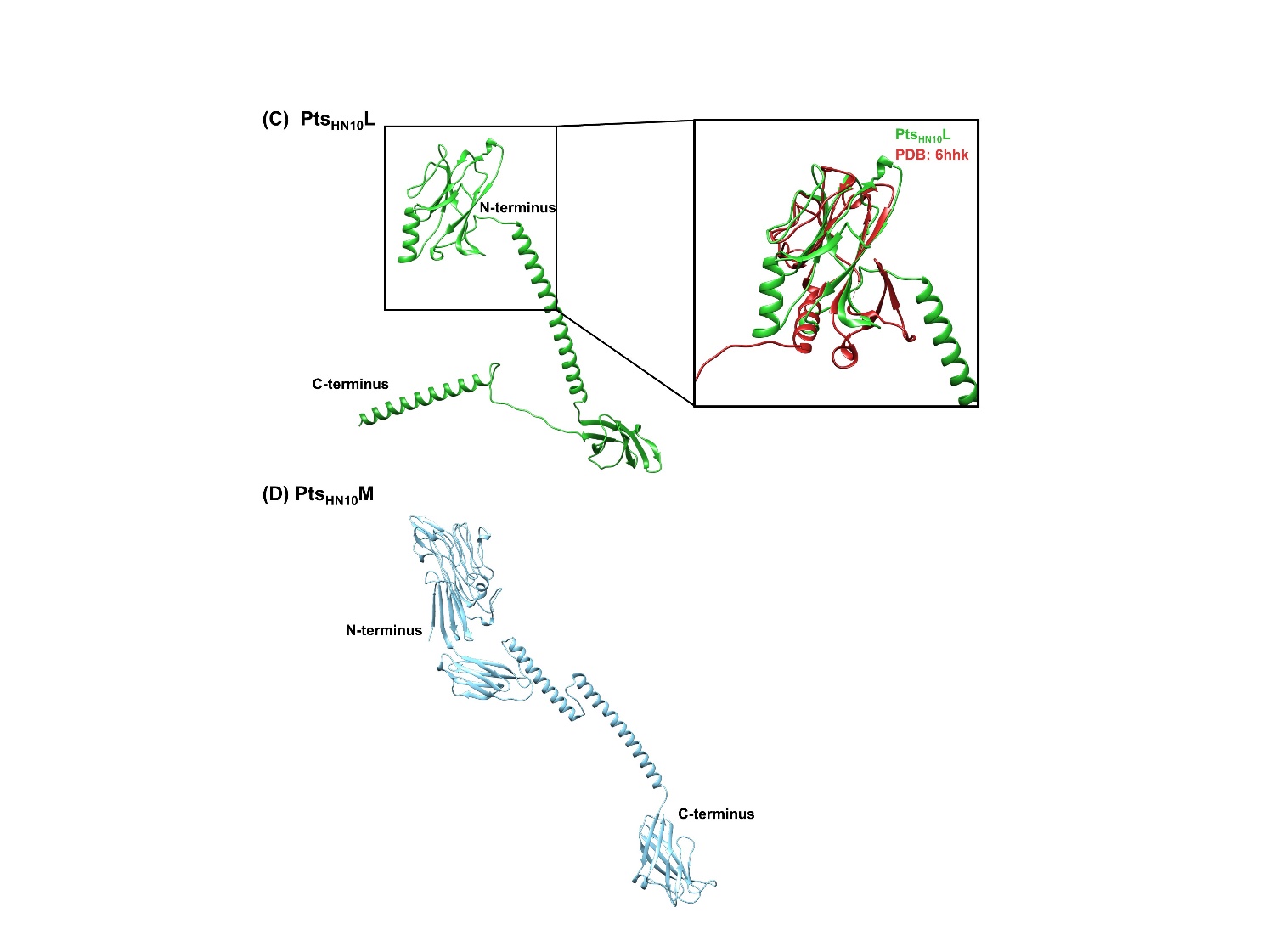


**Supplementary Figure S1.** *In silico* analysis of putative phage RBPs. **(A)** Amino acid sequence alignment of Pts_HN10_L from *C. difficile* strain HN10 (CDHN10) prophage, gp29 from ΦCDMH1 (GenBank accession no. YP_009032171.1), and PtsL from CD630 prophage (AOY11786.1). **(B)** The amino acid sequence alignment of Pts_HN10_M from CDHN10 prophage, gp30 from ΦCDMH1 (YP_009032172.1), and PtsM from CD630 (AOY11787.1). The level of consensus amino acids is shaded from black (identical) to white (no consensus). Domain prediction is indicated in green and blue boxes. Structural prediction of **(C)** Pts_HN10_L and **(D)** Pts_HN10_M was executed using AlphaFold2 (Jumper et al., 2021). The N- and C-terminus of both proteins are indicated. The superposition of the N-terminus of Pts_HN10_L (green) and gp105 of *Listeria* phage A511 (PDB: 6hhk; red) are visualized using ChimeraX (Pettersen et al., 2021).


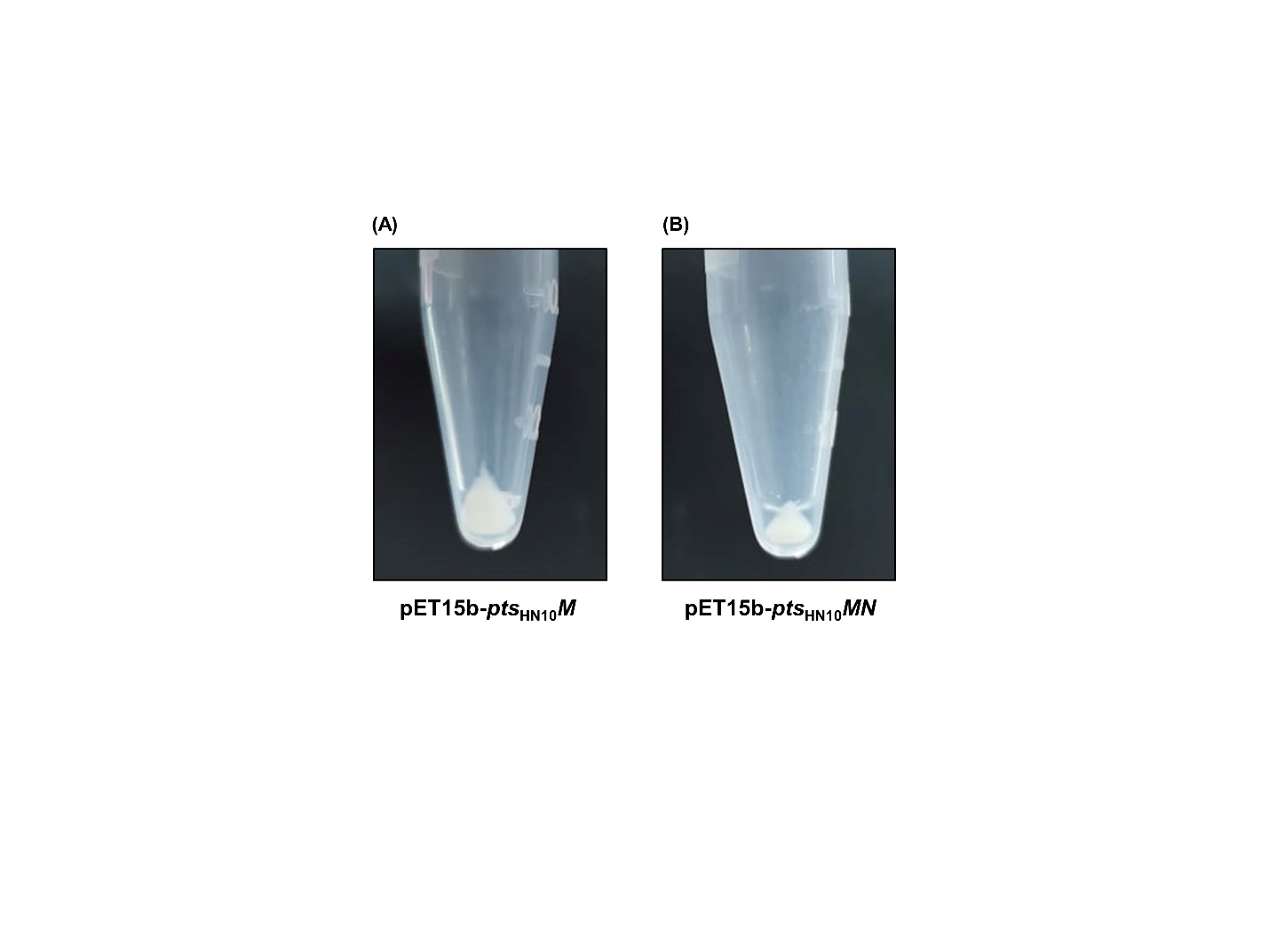
**Supplementary Figure S2.** Purified Pts_HN10_M from **(A)** pET15b-*pts*_HN10_*M* shows more aggregation than that of **(B)** pET15b-*pts*_HN10_*MN* construct. Supernatant fractions of purified proteins from indicated constructs were incubated at 4°C for 16 h and centrifuged at 10,000 x g for 15 min.


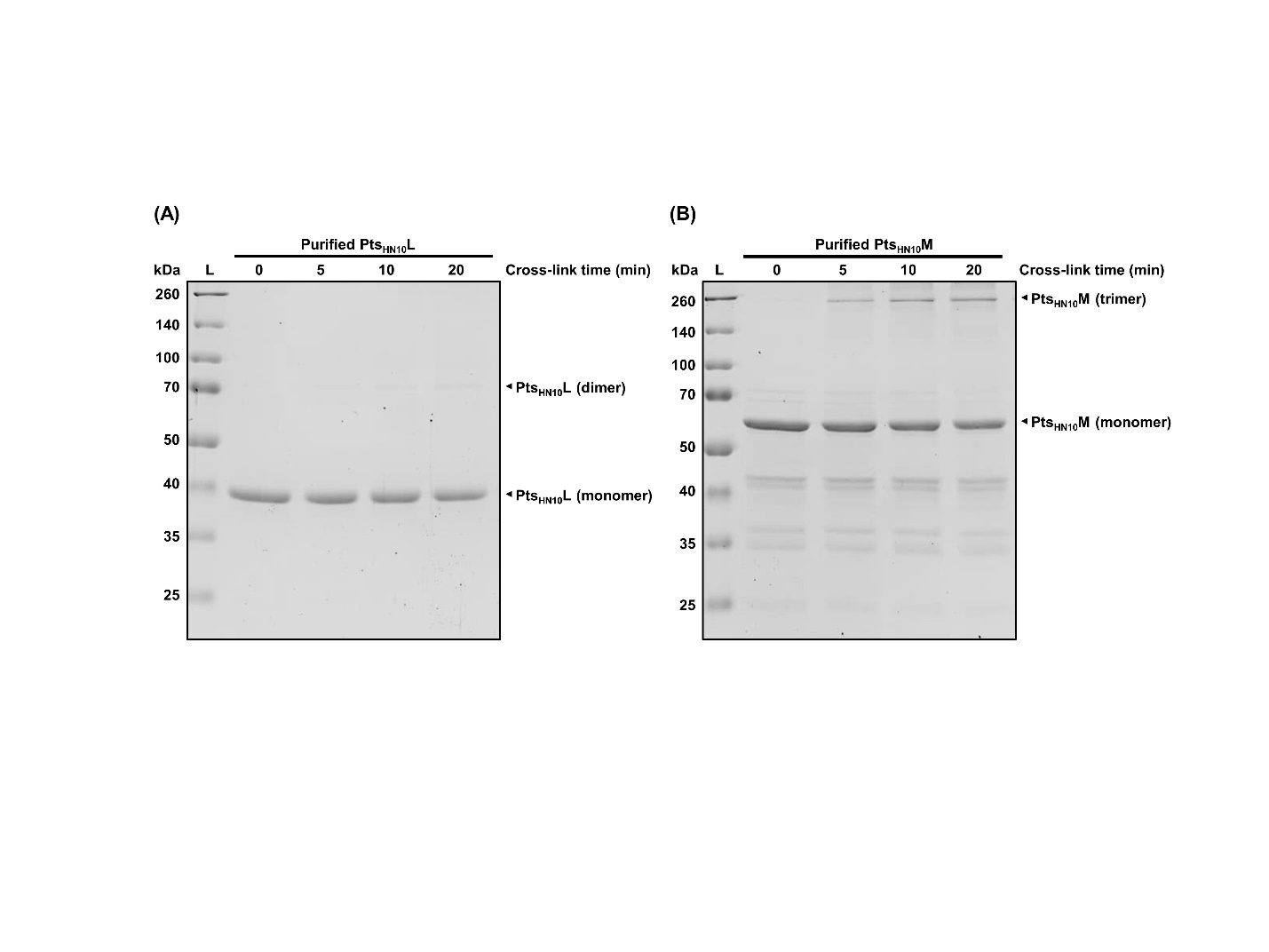
**Supplementary Figure S3.** The cross-linking assay reveals the oligomeric state of purified **(A)** Pts_HN10_L and **(B)** Pts_HN10_M. The incubation times of cross-linking reaction (5, 10, and 20 min) are shown. The estimated oligomeric forms of both Pts_HN10_L and Pts_HN10_M are indicated.


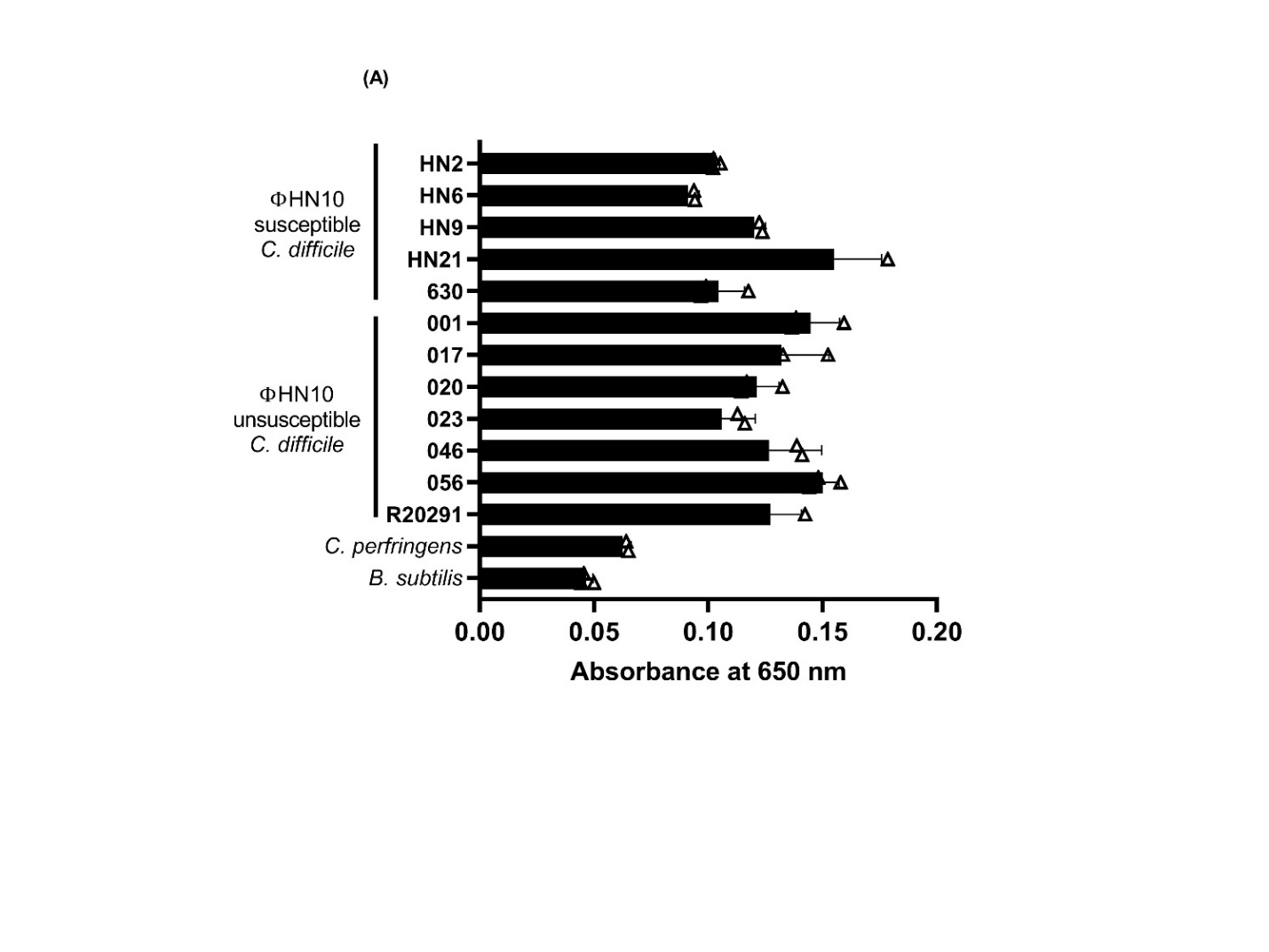


**Supplementary Figure S4.** The binding specificity of Pts_HN10_M is measured by ELISA. The signal of ELISA between purified His_6_-Pts_HN10_M and a group of phage susceptible strains (HN2, HN6, HN9, HN21 and 630) and unsusceptible strains (001, 017, 020, 023, 046, 056, and R20291). The graph shows the mean and standard deviation from three replicates.


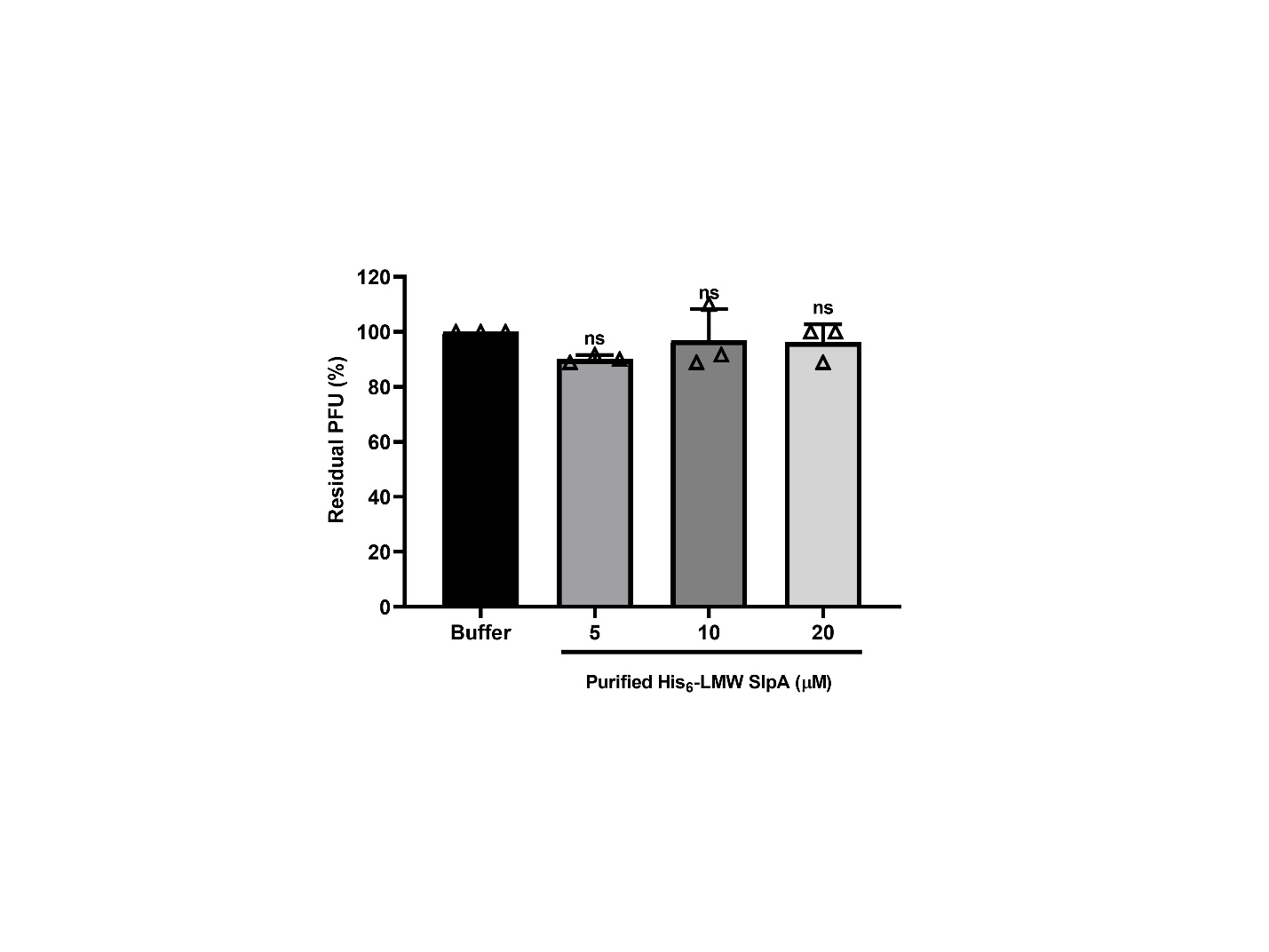


**Supplementary Figure S5.** Phage inactivation assay suggests the LMW SlpA functions as a reversible receptor. Residual PFU percentage after incubation of phage and different concentrations of purified His_6_-LMW SlpA. The graph shows the mean and standard deviation from three replicates. ns, non-significant difference.

## Supplementary Table

**Supplementary Table S1.** Oligonucleotides used in this study

| **Oligonucleotide** | **Sequence (5’-3’)^a^** | |  |
| --- | --- | --- | --- |
| **pET15b_FW** | | *CGAAAGGAAGCTGAG*TTGGCT | |
| **pET15b_RW** | | **CGGATCCTCGAGCAT**ATGGCT | |
| **His_6_-Pts_HN10_L_FW** | | **ATGCTCGAGGATCCG**TTGGCTATAGATAAAAGTTATTACAC | |
| **His_6_-Pts_HN10_L _RW** | | *CTCAGCTTCCTTTCG*CTAACAATAAGTTATCAACTCATTTACACTTG | |
| **His_6_-Pts_HN10_M_FW** | | **ATGCTCGAGGATCCG**ATGCAGACTGAATGGAATTTTAATTATG | |
| **His_6_-Pts_HN10_M_RW** | | *CTCAGCTTCCTTTCG*TTAAATATATATTTTGTAAGTTAATGTAGCTCCC | |
| **Pts_HN10_N_RW** | | *CTCAGCTTCCTTTCG*CTATAAATTGTTATTTAATTTTATATTTTCTACCTCAAAGG | |
| **pDuet-1_His_6__FW** | | *CTGCAGGTCGACAAG*CTTGCG | |
| **pDuet-1_His_6__RW** | | **CGCCGAGCTCGAATT**CGGATC | |
| **pDuet-1_FW** | | *ATCGCTGACGTCGGT*ACCCTC | |
| **pDuet-1_RW** | | **GTGGCCGGCCGATAT**CCAATT | |
| **His_6_-Pts_HN10_L_Duet-1_FW** | | **AATTCGAGCTCGGCG**TTGGCTATAGATAAAAGTTATTACAC | |
| **His_6_-Pts_HN10_L_Duet-1_RW** | | *CTTGTCGACCTGCAG*CTAACAATAAGTTATCAACTCATTTACACTTG | |
| **Pts_HN10_M_1-269_Duet-1_FW** | | **ATATCGGCCGGCCAC**ATGCAGACTGAATGGAATTTTAATTATGCT | |
| **Pts_HN10_M_1-269_Duet-1_RW** | | *ACCGACGTCAGCGAT*TTATGGCAATGCTTGTAGTAATGTTAT | |
| **Pts_HN10_M_1-376_Duet-1_FW** | | **ATATCGGCCGGCCAC**TATGCTAAAATAACATTACTACAAGCATTG | |
| **Pts_HN10_M_1-376_Duet-1_RW** | | *ACCGACGTCAGCGAT*TTATTTAAATCCTGCATTAACCTC | |
| **Pts_HN10_M_1-465_Duet-1_FW** | | **ATATCGGCCGGCCAC**AATTTAAATGATATATCAACAAAATTAGTTGAGG | |
| **Pts_HN10_M_1-465_Duet-1_RW** | | *ACCGACGTCAGCGAT*TTAGTCATATATACTTAAGTTAAAATT | |
| **Pts_HN10_M_1-570_Duet-1_FW** | | **ATATCGGCCGGCCAC**GGAACTTATACATTCACAAAAAC | |
| **Pts_HN10_M_1-570_Duet-1_RW** | | *ACCGACGTCAGCGAT*TTATTAAATATATATTTTGTAAGTTAATGTAGC | |
| **Pts_HN10_M_N-terminus_RW** | | *CTCAGCTTCCTTTCG*TTATGGCAATGCTTGTAGTAATGTTAT | |
| **Pts_HN10_M_C-terminus_FW** | | **ATGCTCGAGGATCCG**TATGCTAAAATAACATTACTACAAGCATTG | |

Jumper, J., Evans, R., Pritzel, A., Green, T., Figurnov, M., Ronneberger, O., et al. (2021). Highly accurate protein structure prediction with AlphaFold. *Nature* 596(7873)**,** 583-589. doi: 10.1038/s41586-021-03819-2.

Pettersen, E.F., Goddard, T.D., Huang, C.C., Meng, E.C., Couch, G.S., Croll, T.I., et al. (2021). UCSF ChimeraX: Structure visualization for researchers, educators, and developers. *Protein Sci* 30(1)**,** 70-82. doi: 10.1002/pro.3943.
